# Cellular immunity against cytomegalovirus and risk of infection after kidney transplantation

**DOI:** 10.3389/fimmu.2024.1414830

**Published:** 2024-06-28

**Authors:** Kjersti B. Blom, Grete B. Kro, Karsten Midtvedt, Trond G. Jenssen, Anna Varberg Reisæter, Hallvor Rollag, Anders Hartmann, Solbjørg Sagedal, Ivar Sjaastad, Garth Tylden, Gro Njølstad, Einar Nilsen, Jon A. Birkeland, Anders Åsberg

**Affiliations:** ^1^ Department of Nephrology, Oslo University Hospital, Ullevål, Norway; ^2^ Institute for Experimental Medical Research, Oslo University Hospital, Ullevål, Norway; ^3^ KG Jebsen Center for Cardiac Research, University of Oslo, Oslo, Norway; ^4^ Faculty of Medicine, University of Oslo, Oslo, Norway; ^5^ Department of Microbiology, Oslo University Hospital, Oslo, Norway; ^6^ Department of Transplantation Medicine, Oslo University Hospital, Oslo, Norway; ^7^ Department of Microbiology and Infection Control, University Hospital of North Norway, Tromsø, Norway; ^8^ Department of Microbiology, Haukeland University Hospital, Bergen, Norway; ^9^ Department of Microbiology, Møre and Romsdal Hospital Trust, Molde, Norway; ^10^ The Norwegian Renal Registry, Oslo University Hospital, Rikshospitalet, Oslo, Norway; ^11^ Department of Pharmacy, University of Oslo, Oslo, Norway

**Keywords:** CMI (cell mediated immunity), cytomegalovirus (CMV), kidney transplansplantation, CMV-IGRA, cytomegalovirus infection

## Abstract

**Introduction:**

Cytomegalovirus (CMV) infection remains a challenge following kidney transplantation (KTx). Currently, CMV-IgG serostatus at transplantation is used to individualize CMV preventive strategies. We assessed the clinical utility of CMV-IGRA for predicting CMV infection following KTx.

**Methods:**

We performed a nationwide prospective cohort study from August 2016 until December 2022. Data from all adult KTx recipients in Norway, n=1,546 (R+; n=1,157, D+/R-; n=260, D-/R-; 129), were included with a total of 3,556 CMV-IGRA analyses (1,375 at KTx, 1,188 at eight weeks, 993 one-year after KTx) and 35,782 CMV DNAemia analyses.

**Results:**

In R+ recipients CMV-IGRA status, measured at any of the time-points, could not identify any differential risk of later CMV infection. D+/R- recipients remaining CMV-IGRA negative 1-year after transplantation (regardless of positive CMV DNAemia and/or CMV IgG status at that time) had increased risk of developing later CMV infection compared to D+/R- recipients who had become CMV-IGRA positive (14% vs. 2%, p=0.01).

**Conclusion:**

Knowledge of pre-transplant CMV-IGRA status did not provide additional information to CMV-IgG serostatus that could improve current post-transplant CMV treatment algorithms. However, D+/R- recipients with a persisting negative CMV-IGRA one-year after transplantation remained at increased risk of experiencing later CMV infection. Therefore we advocate post-transplant CMV-IGRA monitoring in these patients.

## 1 Introduction

Cytomegalovirus (CMV) is one of the most common opportunistic viruses following kidney transplantation (KTx) and warrants close monitoring to initiate preventive strategies for optimizing outcomes (1). The seroprevalence of CMV IgG in kidney transplant recipients and donors vary with geographic region and sex, but is usually in the range of 60–90% in adults (2). Primary CMV infections are most often mild or asymptomatic in immunocompetent hosts, after which CMV establishes latency with periodic asymptomatic reactivation (3). Following KTx, the recipients require life-long immunosuppression leading to increased risk of CMV disease following reactivation or primary infection (4, 5). In KTx, primary CMV infection as well as reactivation may cause significant complications, including acute rejections, graft loss and death (6).

To minimize the impact of post-transplant CMV infection and disease, one of two main preventive strategies is used, preemptive therapy or primary prophylaxis (7). The choice of strategy is based on a risk-stratification, mainly according to donor and recipient CMV immunoglobulin G (IgG) serostatus at time of transplantation as well as the availability of post-transplant CMV DNA monitoring (8). Recipients who are CMV seronegative and receive a kidney from a seropositive donor (D+/R-), have a high risk of developing post-transplant CMV infection and disease, while CMV seropositive (R+) kidney transplant recipients are usually considered to be at intermediate risk, regardless of donor CMV serostatus. D-/R- recipients are considered low-risk patients (9). Antiviral drugs inhibit viral replication, but sufficient control of viral replication is also dependent on the humoral and cellular response of the recipients’ immune system. It is therefore likely that a more personalized stratification that also considers the immunosuppressive state of the KTx patients may potentially improve outcomes.

T-cells are of special importance in the hosts control of CMV infections, where the CD4 T-cells orchestrate the immune response and CD8 T-cells kill infected cells. Activated T-cells produce pro-inflammatory and antiviral cytokines, such as interferon gamma (INF-γ) (3). The production of INF-γ after stimulation with CMV antigens reflects the degree of the patients T-cell response to CMV. CD8 T-cell activity against CMV has been shown to provide additional information on the ability to withstand CMV infections compared to CMV serostatus alone (10–16). In line with this, current international guidelines on management of CMV in solid organ transplantation state that “*data are accumulating that suggest immune monitoring may be considered in combination with viral load monitoring to improve the assessment of the individual`s ability to control CMV” (* 8). Most studies addressing CMV immune monitoring have however only included relatively small number of kidney transplant recipients (10, 11). One method to measure T-cell activity in this setting is the CMV- INF-γ release assay (CMV-IGRA) (8, 17). The CMV-IGRA is currently not available at most microbiology laboratories, but the analysis can be made available to all patients as it preanalytically only requires the assay test tubes and incubation (e.g. 16–24 hour at 37 degrees Celsius). The tubes can be shipped, if long transport centrifugation is preferred, for INF-γ analyses if not performed locally (8, 18).

The aims of the present study were 1) to explore possible changes in CMV-IGRA status among kidney transplant recipients from before transplantation, to eight weeks and one year after transplantation, and 2) to investigate if CMV-IGRA status *per se*, or changes in status, can predict the risk of later CMV infection.

## 2 Materials and methods

### 2.1 Study design and participants

We conducted a historic prospective cohort study of all adult kidney transplant recipients in Norway. A CMV-IGRA test was to be performed at hospital admittance for kidney transplantation as well as eight weeks and one year after transplantation, starting August 2016 (i.e. patients transplanted up to one year before only contributed with an eight-week and/or one-year CMV-IGRA result). In this analysis samples were included from patients transplanted as late as December 2022 and they then only contributed with samples for CMV-IGRA measurements from the time of transplantation (censoring date is December 23^rd^, 2022).

**Figure 1:**
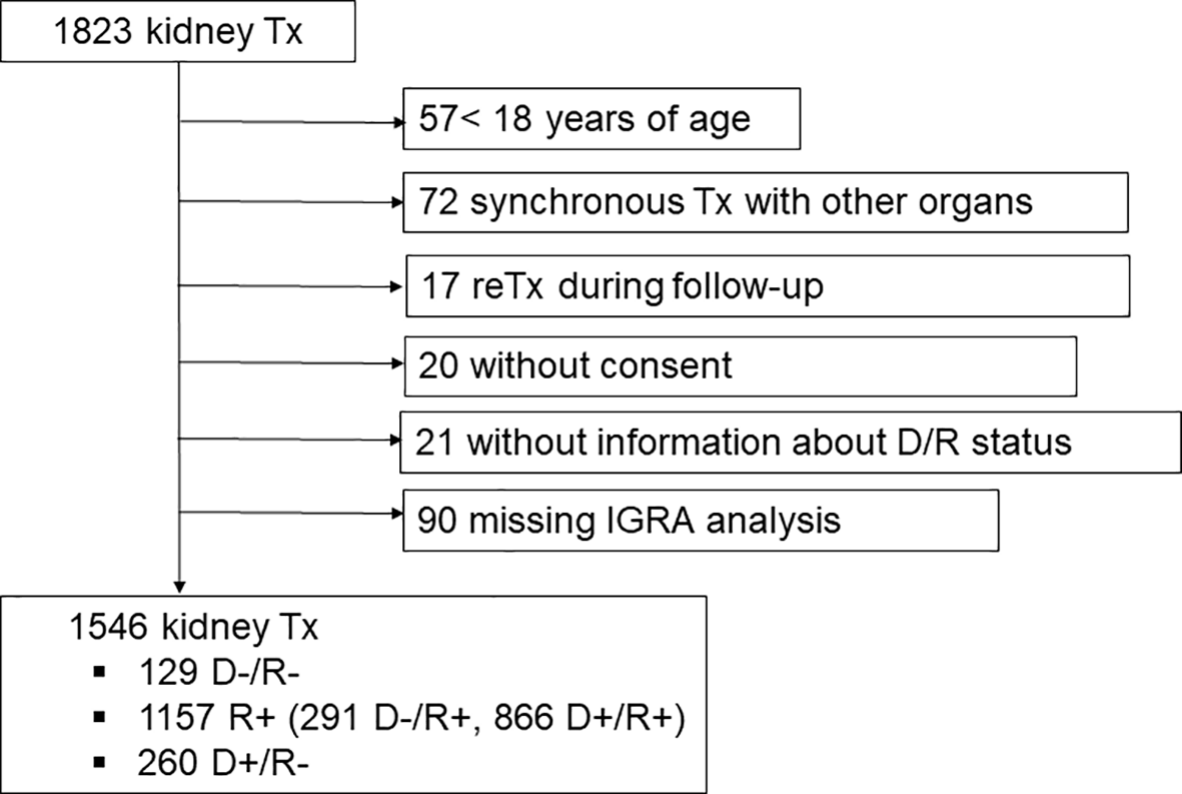
Flow-chart depicting exclusion of ineligible kidney transplant recipients from the study.

Of the 1,823 kidney transplantations performed in the study period, a total of 1,546 (85%) transplantations contributed with samples in the analysis (**Figure 1**). Reasons for exclusion from the analyses were: 57 were transplantations in children <18 years, 72 were synchronous transplantations with other organs, 17 were the second transplantation for a recipient in the study period, 20 patients had not provided consent to perform research on their samples, D/R CMV serostatus was lacking in 21 cases and CMV-IGRA analyses were missing in 90 transplantations. The study was approved by the Regional Ethics Committee of south-east Norway (REK 43147).

### 2.2 CMV T-cell activity and IgG

CMV-IGRA (QuantiFERON, Qiagen) is an established method for measuring specific CD8 T-cell activity against CMV in a clinical setting (17) and was introduced at Oslo University Hospital as a routine analysis in kidney transplant recipients in August 2016. The method is described in detail in the **Supplementary File**.

For the data analyses only samples with a value greater than two times the cut-off (>0.4 IU INF-γ/mL) were considered “positive”, whereas weakly positive, grey-zone reactive and negative results were considered “negative”. Analyses with inconclusive results, e.g. positive- and/or negative controls that did not pass the method criteria, were treated as “missing” in the present statistical analyses, however, an overview of the distribution of inconclusive results is shown in **Supplementary Table 1**. No sub analyses were performed with these samples as a separate group due to few samples.

The CMV-IgG serostatus of all recipients at transplantation were centrally analyzed at the Department of Microbiology at the transplantation center using Architect CMV IgG, Abbot. Donor CMV-IgG serostatus was assessed by the respective transplant center in the Scandiatransplant consortium and reevaluated at the Norwegian transplant center in case of missing information.

### 2.3 CMV-DNAemia analyses

All seven laboratories performing CMV-DNA analyses in Norway provided data to the current study, and the respective methods have been described previously (19). In short, all laboratories used real-time quantitative PCR analyses for quantitation of CMV-DNA, mainly in plasma (<2% in whole blood). Results are reported as copies/mL or international units (IU)/mL. The difference between copies/mL and IU/mL is not considered significant in the analyses as the conversion factor is 1.1 (20). CMV infection was defined as detection of CMV-DNAemia ≥ 600 IU/ml plasma regardless of symptoms. In samples analyzed at Oslo University Hospital after March 3^rd^ 2021, the limit was ≥1000 IU/mL plasma, due to a change in the quantitative PCR method (19).

### 2.4 CMV preventive strategies

From the day of transplantation, CMV intermediate risk recipients (R+) and CMV low-risk recipients (D-R-) were followed by a preemptive CMV strategy. So-called CMV high-risk recipients (D+/R-) received once-daily valganciclovir prophylaxis (900 mg, dose adjusted according to renal function) for six months post-transplantation. All recipients, both R+ and D-/R- preemptive patients, and D+/R- patients, were subject to at least weekly CMV-DNA quantitation during the first two months post-transplant and monthly thereafter up to one-year post-transplant. After the first post-transplant year, CMV-DNA quantification was performed on clinical indication.

In R+ recipients treatment with valganciclovir (900 mg twice daily, dose adjusted to renal function) was initiated in case of CMV-DNAemia above 600 IU/mL plasma (1000 IU/mL plasma from March 2021 at the Oslo University Hospital laboratory due to adjustment in the quantitative method). Length of valganciclovir treatment was at least three weeks, or until two negative CMV-DNAemia measurements separated by at least one week were attained.

### 2.5 Immunosuppressive protocol

Recipients with standard immunological risk received induction treatment with methylprednisolone and IL-2 receptor antibody (basiliximab), and maintenance treatment with glucocorticoids (5 mg/day), the cell proliferation inhibitor mycophenolate, and a calcineurin inhibitor (CNI); either tacrolimus (Tac) (target trough concentrations 4–7 µg/L from the day of engraftment) (21) or cyclosporine (CsA) (the target was initially 200–300 µg/L tapering to 75–125 µg/L from 6 months). Tac was preferred for all patients except for patients with impaired glucose tolerance. Tac was combined with 750 mg mycophenolate mofetil (540 mg mycophenolate sodium) twice daily, while CsA was combined with 1000 mg mycophenolate mofetil (720 mg mycophenolate sodium) twice daily. Prednisolone was tapered from 20 mg/day to 10 mg/day by the second month and further to 5 mg/day from month six.

Patients classified as immunological intermediate-risk, i.e. patients with an ABO blood-type incompatible (ABOi) transplant, panel reactive antibodies (PRA) >20% or immunological high-risk (known donor specific antibodies (DSA) at the time of transplantation) had higher CNI targets. Tac trough targets were 10–12 µg/L the first month, 6–10 µg/L the second month (ABOi) or the first year (PRA positive/DSA positive) and 5–8 µg/L from the third month (ABOi) or after the first year (PRA positive/DSA positive), respectively. Corresponding CsA trough targets were; 250–350 µg/L, 150–250 µg/L and 100–175 µg/L. As induction therapy they also received methylprednisolone in combination with either rituximab, basiliximab and intravenous human immunoglobulin (ABOi/DSA positive) or ATG (PRA positive).

### 2.6 Statistical considerations

Incidence of infections were compared by applying crude Kaplan Meyer survival analyses and the log-rank test with the “survival” and “survminer” R-packages (R version 4.2.2). Censoring date was December 23^rd^, 2022. Results were considered statistically significant when the p-value was equal to or below 0.05.

## 3 Results

A total of 1,546 patients were subjected at least one CMV-IGRA test. The demographic characteristics at time of transplantation of these 1,546 patients are shown in **Table 1**. In total, 3,556 CMV-IGRA analyses were performed at transplantation (N=1,375), at eight weeks (N=1,188) and one year (N=993) after transplantation. The CMV serostatus risk group at transplantation was R+ in 1,157 (D-/R+ 291, D+/R+ 866), D+/R- in 260 and D-/R- in 129 patients. The median follow-up time post-transplantation was 3.5 years (range 15 days to 6.4 years). The distribution of the CMV-IGRA analyses between pretransplant CMV serostatus risk groups at the different time points is shown in **Table 2**. All 35,782 CMV-DNAemia analyses performed in these patients are included in the analysis. At least one CMV infection was detected in 25% of the patients that were R+ at transplantation, 33% of the D+/R- patients, and 2% of the D-/R- patients (p<0.001) during the follow-up period (**Supplementary Figure 1**).

**Table 1:** Demographic data at time of kidney transplantation.

Characteristics	N = 1,546
Age, years	55.0 ± 14.7
Male sex, n (%)	961 (62%)
Donor/Recipient CMV serostatus: R+ D+/R- D-/R-	1,157 (75%) 260 (17%) 129 (8%)
BMI, kg/m^2^	25.9 ± 4.6
Hypertension*, n (%)	1436 (93%)
Pretransplant diabetes, n (%)	296 (19%)
Active smoker, n (%)	206 (13%)
Living donor kidneys, n (%)	393 (25%)
Donor age, years	51.8 ± 17.0
HLA AB mismatches	2.2 ± 1.1
HLA DR mismatches	1.0 ± 0.7
Preemptive transplants, n (%)	356 (23%)
Retransplants, n (%)	242 (15%)

*Blood pressure above 130/80 mmHg and/or use of at least one antihypertensive drug. SD, standard deviation; n, number; BMI, body mass index. Data are presented as mean ± SD or numbers (%).

**Table 2:** CMV-IGRA status in respective donor/recipient risk groups according to pretransplant CMV-IgG serostatus; pretransplant, eight weeks and one year after kidney transplantation.

Pretransplant risk group	CMV-IGRA negative	CMV-IGRA positive
Pre transplant (n=1,375)		
R+ (n=1,035)	160 (15%)	875 (85%)
D+/R- (n=232)	232 (100%)	0 (0%)
D-/R- (n=108)	108 (100%)	0 (0%)
Eight weeks (n=1,188)		
R+ (n=884)	135 (15%)	749 (85%)
D+/R- (n=203)	203 (100%)	0 (0%)
D-/R- (n=101)	101 (100%)	0 (0%)
One year (n=993)		
R+ (n=738)	79 (11%)	659 (89%)
D+/R- (n=168)	106 (63%)	62 (37%)
D-/R- (n=87)	84 (97%)	3 (3%)

Data are presented as numbers (%).

For the data analyses only positive samples (>0.4 IU INF-γ/mL) were considered “positive”. Weakly positive, grey-zone reactive and negative results were considered “negative”.

### 3.1 CMV-IGRA status related to CMV reactivation, or reinfection, during follow-up in R+ patients

**Figure 2:**
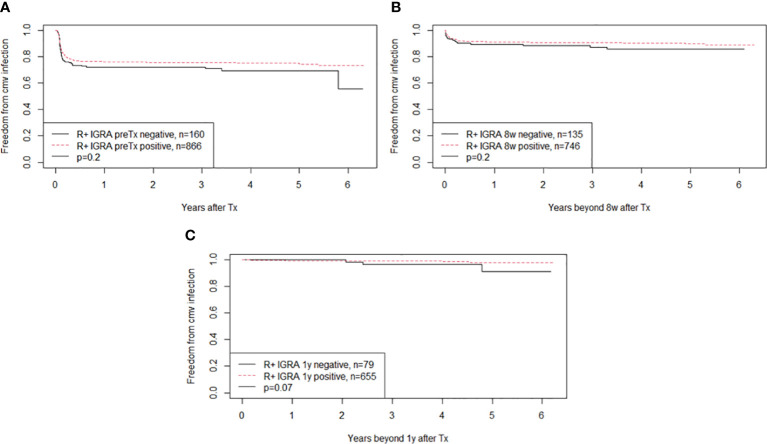
The figure shows Kaplan-Meier analysis of freedom from CMV infection after transplantation in CMV seropositive kidney recipients (abbreviation R+). The curves show patients with positive or negative CMV-IGRA status at time of transplantation in **(A)**, IGRA status at 8 weeks in **(B)** and at 1 year in **(C)**. Patients were subjected to weekly CMV DNAemia monitoring during the first two months, monthly up to one year and on clinical indication thereafter. Monitoring of CMV were lacking in nine, three and four patients respectively in **(A–C)**.

CMV-IGRA status at time of transplantation was available in 1,035 R+ patients (90%). Of these, 875 (85%) were CMV-IGRA positive and 160 (15%) were CMV-IGRA negative (**Table 2**). 33% of the 160 CMV-IGRA negative R+ patients used immunosuppressive drugs before transplantation, 53% of these due to previous transplantations. One (<1%) of the R+ patients was CMV-IGRA inconclusive (**Supplementary Table 1**). Among the 866 R+ recipients with a positive CMV-IGRA at transplantation, 24% developed CMV reactivation or reinfection. This was not significantly different from 29% of the R+ recipients who were CMV-IGRA negative at transplantation (**Figure 2A**, p= 0.2).

The CMV-IGRA status eight weeks after transplantation was available in 884 R+ patients (**Table 2**). Five patients (1%) were CMV-IGRA inconclusive (**Supplementary Table 1**). Among the patients with a positive CMV-IGRA at this time-point (n=746), later CMV reactivation or reinfection occurred in 10%. This was not significantly different from later CMV infection in recipients that were CMV-IGRA negative eight weeks after transplantation (n=135) for which CMV reactivation or reinfection occurred in 13% (**Figure 2B**, p=0.2).

**Figure 3:**
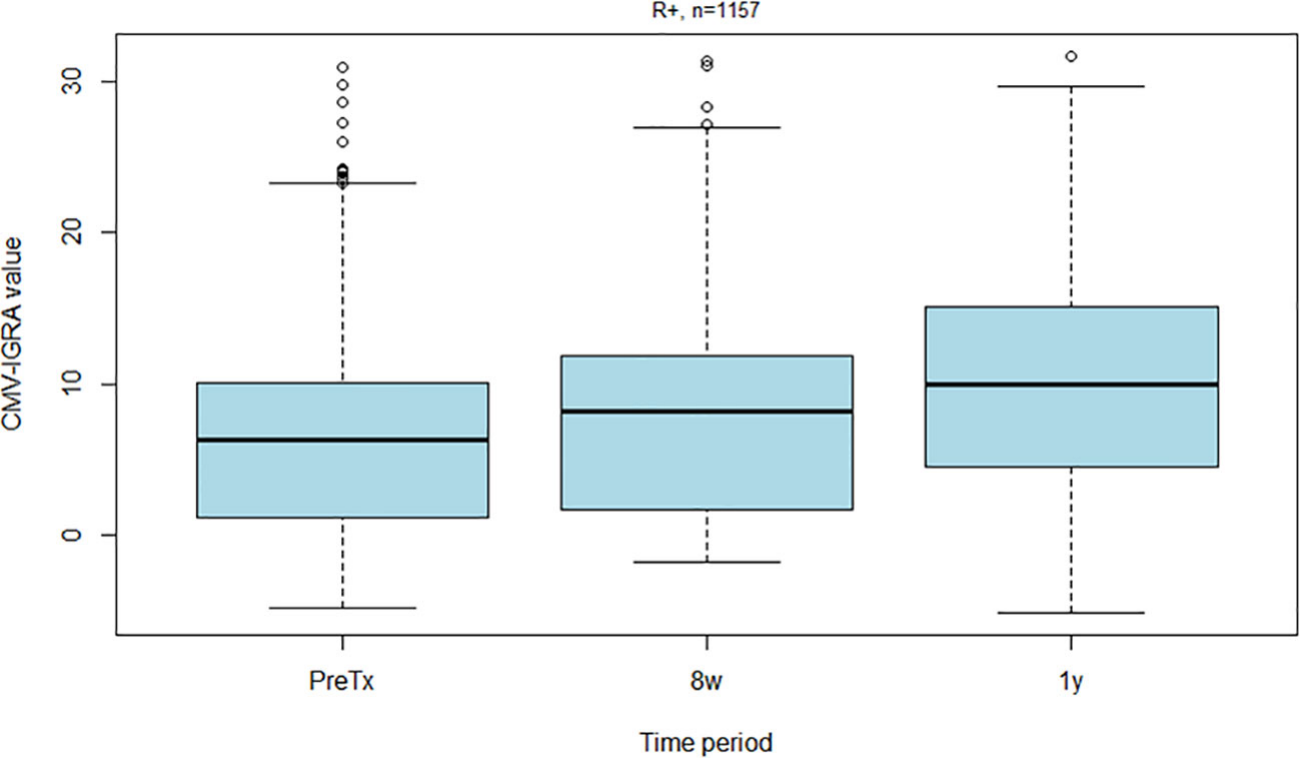
Box plot showing CMV-IGRA values among all CMV seropositive kidney recipients before transplantation, eight weeks post-transplantation and one year post-transplantation.

**Figure 4:**
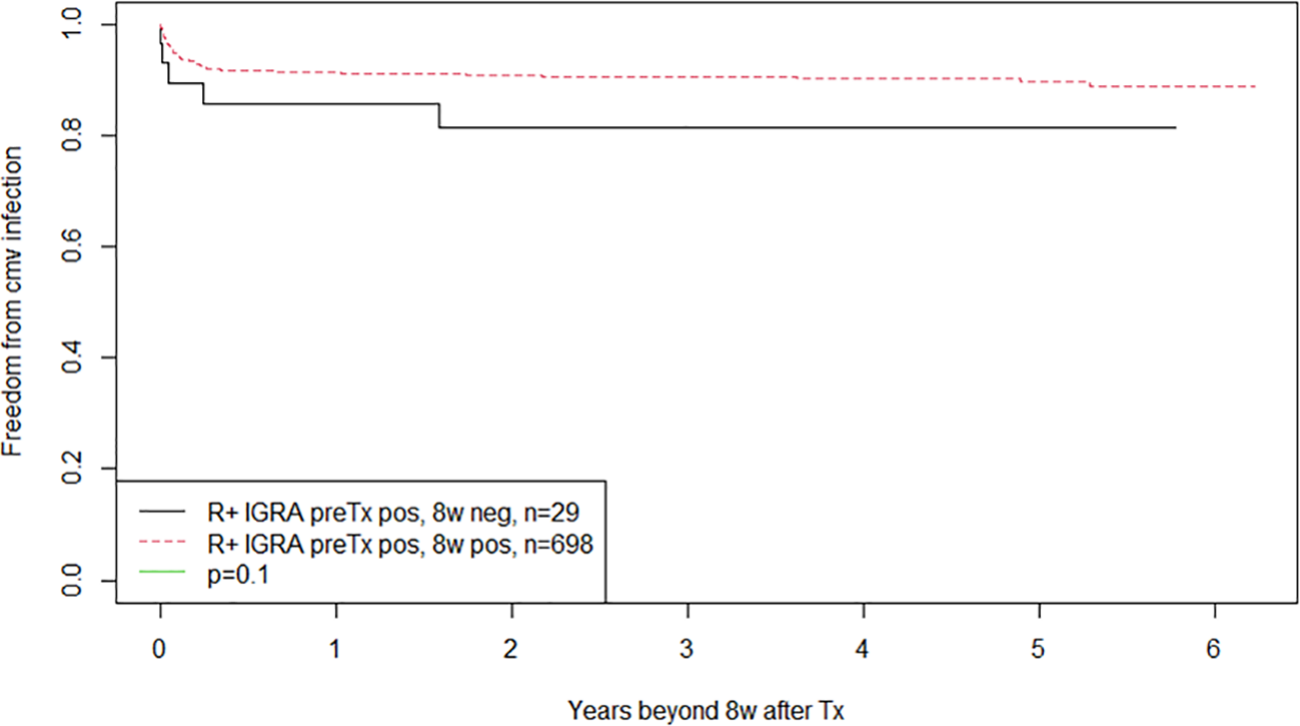
Kaplan-Meier analysis of freedom from CMV infection after eight weeks posttransplant. Patients converting from positive to negative CMV-IGRA status eight weeks after transplantation are compared to those maintaining positive CMV-IGRA status in CMV seropositive kidney recipients. All patients were subjected to monthly CMV DNAemia monitoring up to one year and on clinical indication thereafter.

CMV-IGRA results at the three different time points for R+ recipients are shown in **Figure 3**. Those R+ recipients who changed from a CMV-IGRA positive status at transplantation to a negative CMV-IGRA status eight weeks after transplantation (n=29) experienced a non-significant trend towards more CMV infections (17%) compared to the 698 R+ recipients who remained CMV-IGRA positive (9%) (p = 0.1) (**Figure 4**).

After one year, 89% of the R+ patients were CMV-IGRA positive versus 85% before, as well as eight weeks after transplantation (**Table 2**). One (<1%) was CMV-IGRA inconclusive (**Supplementary Table 1**). A negative CMV-IGRA status in R+ recipients one year after transplantation showed a non-significant trend towards increased late CMV infections (4%) compared with CMV-IGRA positive patients at this time-point (1%) (p=0.07) (**Figure 2C**).

The patients with inconclusive CMV-IGRA at eight weeks post-transplantation (n=5) seem to have a numerically increased risk of CMV infection compared to the other R+ recipients (figure not shown).

### 3.2 CMV-IGRA status related to primary CMV infection during follow-up in R- patients

**Figure 5:**
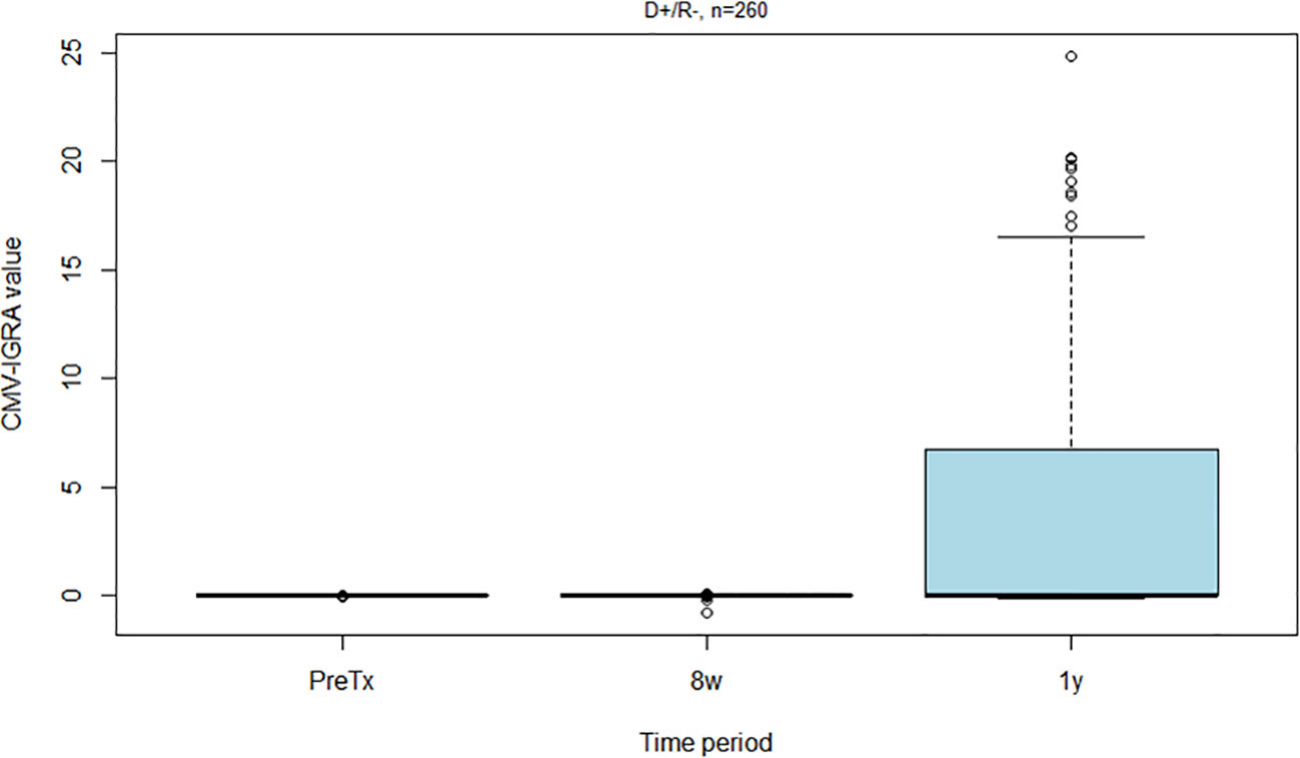
Box plot showing CMV-IGRA values among all CMV negative kidney recipients who received a CMV positive kidney (D+/R-) before transplantation, eight weeks post-transplantation and one year post-transplantation.

The CMV-IGRA status was available in 340 R- patients at the time of transplantation (**Table 2**). All D-/R- and D+/R- were CMV-IGRA negative showing full agreement between CMV-IgG serostatus and CMV-IGRA status in pretransplant CMV naïve patients. CMV-IGRA results at the three different time points for D+/R- recipients are shown in **Figure 5**, and in **Supplementary Figure 2** for D-/R-.

Patients who are D-/R- are at low-risk of CMV infection and disease, nevertheless, they may suffer community acquired primary CMV infection. In the 108 D-/R- patients with measured CMV-IGRA only three became positive the first year after transplantation (**Supplementary Figure 2**). CMV-DNAemia was detected in two of these in this time-period.

**Figure 6:**
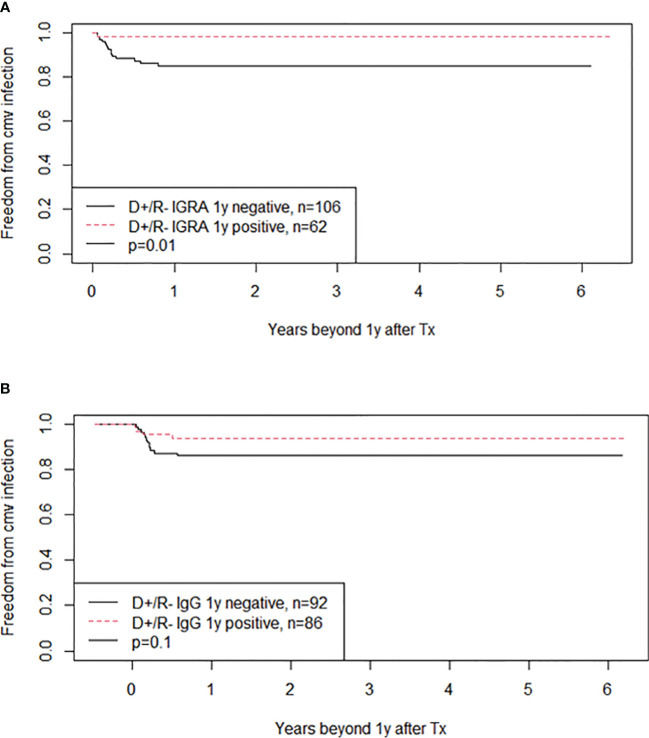
The figure shows Kaplan-Meier analyses of freedom from CMV infection beyond one year posttransplant in CMV negative kidney recipients who received a CMV positive kidney (D+/R-). **(A)** Compare patients with negative or positive CMV-IGRA status at one year after transplantation whereas **(B)** compare patients with negative or positive CMV IgG status one year after transplantation. CMV DNAemia was taken on clinical decisions. The curves are significantly different in **(A)** (p=0.01) but not in **(B)** (p=0.1).

**Figure 7:**
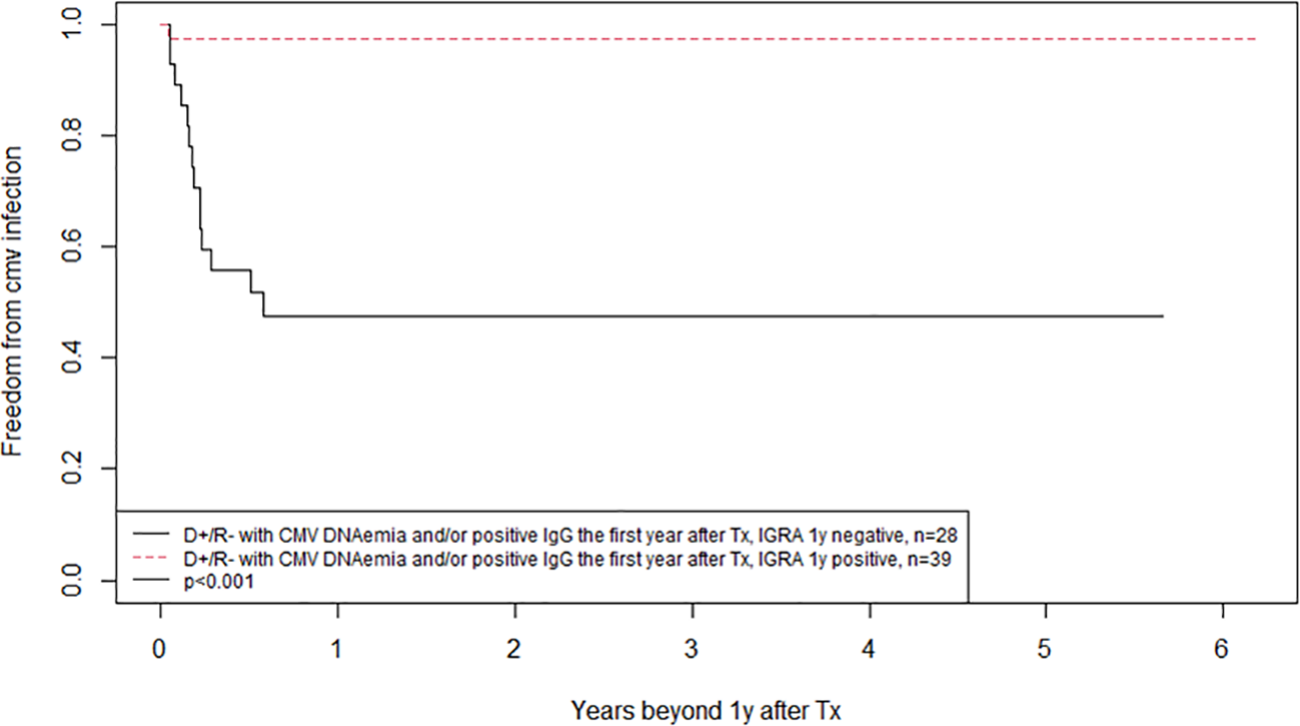
Kaplan-Meier analysis of freedom from CMV infection after one year posttransplant, comparing patients with positive and negative CMV-IGRA status one year after transplantation in CMV negative kidney recipients that received a CMV positive kidney (D+/R-) with a detected CMV infection during the first year post-transplantation (either positive CMV DNAemia and/or positive CMV IgG). Patients were subjected to CMV DNAemia on clinical indication.

Kidney transplant recipients who are D+/R- are at high risk of developing CMV infection after transplantation and therefore receive valganciclovir prophylaxis for six months according to our protocol. None of the 232 D+/R- patients were CMV-IGRA positive at transplantation or eight weeks after (during valganciclovir prophylaxis) (**Figure 5**). However, seven (3%) experienced a CMV infection in the prophylaxis period between transplantation and the CMV-IGRA test performed at eight weeks. Peak CMV DNA values ranged from 1900 to 1,528,000 IU/mL. Also, 62 of the D+/R- kidney transplant recipients (27%) had become CMV-IGRA positive one year after transplantation; preceding CMV-DNAemia had been demonstrated in 38 (61%) of these patients. In addition, 38 of these 62 patients (61%) also showed CMV-IgG seroconversion by 1-year post-transplant. However, among the 38 D+/R- patients with CMV-IgG seroconversion by one-year post-transplant, only 18 (47%) had previously demonstrated CMV-DNAemia. Those D+/R- recipients in whom the CMV-IGRA remained negative one year after transplantation showed a significantly increased risk of later developing CMV infection, 14% in the CMV-IGRA negative versus 2% in the CMV-IGRA positive patients (**Figure 6A**, p=0.01). Also, D+/R- recipients with a demonstrated CMV infection during the first year (positive CMV-DNAemia and/or CMV-IgG seroconversion) but with a persistently negative CMV-IGRA status one year after transplantation showed an increased risk of later developing CMV infection. In the persistent CMV-IGRA negative group 50% (14/28) developed CMV infection versus 3% (1/39) in the CMV-IGRA positive group (**Figure 7**, p<0.001). Those D+/R- patients who had not CMV-IgG seroconverted during the first year after transplantation did not show increased risk of later developing CMV infection compared to seroconverted D+/R- one year after transplantation (**Figure 6B**, p=0.1).

## 4 Discussion

We describe changes in T-cell immune activity against CMV, assessed by CMV-IGRA, from before transplantation and up to one year after kidney transplantation in 1,546 recipients. The main finding is that CMV-IGRA immune monitoring does provide some additional, clinically relevant, information about the risk of later CMV infection in high-risk patients (D+/R-) compared to CMV-IgG seroconversion and intense CMV-DNA monitoring. Pretransplant CMV-IGRA status did not provide any additional risk information to CMV-IgG serostatus regarding later CMV infection. However, in the high-risk CMV serostatus group (D+/R-) with a persistent negative CMV-IGRA status one year after transplantation showed an increased risk of later CMV infection. Demonstration of seroconversion, or previous CMV-DNAemia, was not sufficiently able to exclude this increased risk. Actually, D+/R- patients with detected CMV infection (positive CMV PCR and/or CMV IgG) during the first-year post-transplant, but with a persistent negative CMV-IGRA status showed an increased risk of CMV infection after the first year. In these situations, CMV-IGRA information may improve clinical risk stratification of kidney transplant recipients and potentially improve long-term outcomes, e.g. continued intensive CMV DNAemia monitoring in CMV-IGRA negative patients also after the first year.

To our knowledge, this is the largest study so far on this topic, as previous studies only included a limited number of patients (12–326 transplant recipients) (10–12, 18, 22–27). As expected, and in line with most previous studies (but not all) (12), none of the R- recipients were CMV-IGRA positive before transplantation (23, 24, 27). However, 15% of the R+ recipients were CMV-IGRA negative before transplantation. This is in line with other studies reporting negative pretransplant CMV-IGRA status in 23–50% of R+ recipients (10, 11, 22–24), and 18% CMV-IgG/IGRA discordance in healthy individuals (28). Discordance between CMV serostatus and cellular immunity observed in the current study may be partly due to ongoing immunosuppressive therapy, either treatment for immunological diseases or to avoid development of donor specific antibodies (DSA) while waiting for a retransplant. This could explain some of the cases in our cohort. However, for 67% we could not find any plausible clinical explanation. These patients may be so-called low responder individuals. Healthy CMV-seropositive individuals with negative CMV-IGRA have previously been shown to have weaker humoral and cellular responses to CMV (28). In addition, 50 patients (31%) in the CMV-IGRA negative group were not totally negative, but had a weak T-cell reaction to CMV.

None of the D+/R- kidney transplant recipients developed a positive CMV-IGRA during the first eight weeks post-transplantation. However, 3% developed significant CMV infection in the prophylaxis period before the CMV-IGRA tests at eight weeks were performed. Thus, it seems CMV-IGRA were negative despite a high level of CMV replication in some patients. A possible reason could be the elevated levels of immunosuppression in D+/R- kidney transplant recipients throughout the prophylaxis period, which might compromise their ability to generate a T-cell response.

In our patients, pretransplant CMV-IGRA status did not provide any significant additional information about the risk of later CMV infection compared to conventional serostatus. This finding is in line with a recent study on 55 R+ recipients showing a non-significant trend towards a lower CMV infection rate in pretransplant CMV-IGRA positive recipients (29). However, several previous studies have shown prognostic significance of pretransplant CMI in predicting later CMV events (23, 30, 31). Cantisán et al. (23) reported increased post-transplantation CMV replication among R+ recipients with a negative pretransplant CMV-IGRA (p=0.02). However, the study was conducted in lung- and kidney transplant recipients. Also, as opposed to us, they used a CMV-IGRA cut-off value of >0.2 INF-γ/mL. Both Jarque et al. and Bestard et al. used ELIspot assays to detect CMV specific T-cell immunity. ELIspot assays are highly sensitive, and quantify the frequency of both CD4+ and CD8+ cells that produce INF-γ in response to CMV (32). Still, ELIspot assays require more specialized equipment, which may hinder their wider implementation (33).

Five R+ kidney transplant recipients had inconclusive CMV-IGRA results at eight weeks post-transplantation due to low positive control. As these patients had increased risk of later CMV infection, inconclusive CMV-IGRA due to low positive control might represent severe immunosuppression. However, as the numbers are small, we did not perform survival analysis in this group.

The present analyses suggests that determination of cellular immunity against CMV can further differentiate the post-transplant risk of future CMV infection compared to CMV serology alone in the D+/R- patient groups. The D+/R- patients that had converted to CMV-IGRA positive one year after transplantation showed a lower risk of developing future CMV infection compared to persistent CMV-IGRA negative patients in the current analysis. This difference in risk was not possible to exclude by demonstration of CMV-IgG seroconversion and or CMV-DNAemia episodes during the first year. Including CMV-IGRA monitoring in these high-risk patients, for example in the period after ending prophylaxis, might further individualized the need for continued intensive CMV-DNAemia monitoring.

The main strength of the present analysis is the unselected and large number of patients included, no loss to follow-up, and complete availability of all CMV-DNAemia measurements performed after transplantation (intensive monitoring). Although a total of seven laboratories performed these analyses around the country, all samples the first two months after transplantation, and the majority (87%) of all samples, were analyzed at the laboratory at the national transplant center. Also, since we only assessed if CMV infection occurred, differences between laboratories will be of minor relevance. Additionally, all the CMV-IGRA analyses were analyzed at the national transplant center laboratory. Information about development of CMV disease is, however, unfortunately not available in the present analysis.

*In conclusion*, our study provides a longitudinal overview of T-cell immunity against CMV for patients before and after kidney transplantation. The study supports previous results from smaller studies that repeated CMV-IGRA measurement after transplantation provides additional information to CMV-IgG serology and intensive CMV DNAemia alone on future risk of CMV infection in specific sub-groups of patients (10, 18, 24–26). A one-year assessment of CMV-IGRA in D+/R- patients, seems valuable.

## Data availability statement

The original contributions presented in the study are included in the article/**Supplementary Materials**, further inquiries can be directed to the corresponding author/s.

## Ethics statement

The studies involving humans were approved by Regionale komiteer for medisinsk og helsefaglig forskningsetikk, nr: 43147 Finn Wisløff Finn Skre Fjordholm. The studies were conducted in accordance with the local legislation and institutional requirements. The participants provided their written informed consent to participate in this study.

## Author contributions

KB: Conceptualization, Formal analysis, Writing – original draft, Writing – review & editing. GK: Conceptualization, Data curation, Investigation, Methodology, Project administration, Writing – original draft, Writing – review & editing. KM: Writing – original draft, Writing – review & editing. TJ: Writing – original draft, Writing – review & editing. AR: Writing – original draft, Writing – review & editing. HR: Writing – original draft, Writing – review & editing. AH: Writing – original draft, Writing – review & editing. SS: Writing – original draft, Writing – review & editing. IS: Writing – original draft, Writing – review & editing. GT: Data curation, Investigation, Writing – original draft, Writing – review & editing. GN: Data curation, Investigation, Writing – original draft, Writing – review & editing. EN: Data curation, Investigation, Writing – original draft, Writing – review & editing. JB: Conceptualization, Methodology, Supervision, Writing – original draft, Writing – review & editing. AÅ: Conceptualization, Data curation, Formal analysis, Investigation, Supervision, Writing – original draft, Writing – review & editing.
