# Supplementary material for: Cellular immunity against cytomegalovirus and risk of infection after kidney transplantation
Source: Front Immunol. 2024 Jun 28;15:1414830. doi: 10.3389/fimmu.2024.1414830 (PMC11239502; doi:10.3389/fimmu.2024.1414830)
Supplement: Supplementary file 1 [file DataSheet_1.docx]

**Supplementary text**

*Description of CMV IGRA assay*

The CMV INF-γ release assay (CMV-IGRA, QuantiFERON-CMV, Qiagen) has been established as a method for measuring specific CD8 T-cell activity against CMV in a clinical setting^1^. The test was performed according to the instructions of the manufacturer.

Peripheral blood samples (1ml) was collected in the three QuantiFERON-CMV Blood Collection Tubes according to standard vacutainer sampling. The three tubes were; 1) Negative control tube containing an anticoagulant alone 2) Positive mitogen control tube containing a general T-cell activator, phytohemagglutinin 3) Test tube containing HLA-I restricted CMV peptide epitopes that stimulate a CD8 T-cell response and INF-γ production in CMV-specific CD8 T-cells. The three tubes were shaken and then incubated at 37 °C for 16-24 hours followed by centrifugation at 2,200 g for 10 minutes.

The levels of INF-γ (IU/ml) were thereafter determined in the plasma. Until November 2020 INF-γ was analyzed with QuantiFERON-TB Gold, Qiagen, using BEP 2000. From November 2020 the analysis was changed to an automated platform; Liaison, DiaSorin, using Liaison QuantiFERON-TB Gold Plus kit, DiaSorin, Ireland.

The test result was calculated by subtracting the INF-γ level in the negative control tube from the level in the test tube. The recommended cut-off according to the producer was 0.2 IU/ml, and additionally the result must be ≥ 25% of the negative control. The gray-zone around the cut-off was based on the variation coefficient; cut-off ± (2 x variation coefficient % x cut-off). Results above grey-zone, but less than 2 times cut-off were considered weakly positive. Results higher than 2 times cut-off, that is > 0.4 IU/ml, were positive.

In accordance with the instructions from the manufacturer the analysis was considered indeterminate if the result was negative and there was a lack of general T-cell response, represented by positive mitogen control subtracted negative control being <0.5 IU/ml. Samples with a negative control above 8.0 IU/mL were also considered indeterminate.

**References**

1. Walker S, Fazou C, Crough T, et al. Ex vivo monitoring of human cytomegalovirus-specific CD8+ T-cell responses using QuantiFERON-CMV. *Transpl Infect Dis*. Jun 2007;9(2):165-70. doi:10.1111/j.1399-3062.2006.00199.x

**Table 1:** CMV-IGRA using categorical results in respective Donor/Recipient risk groups according to pre-transplant CMV-IgG serostatus; pre-transplant, eight weeks and one year after kidney transplantation. Data are presented as numbers (%).

|  | **CMV-IGRA negative** | | **CMV-IGRA grey-zone reactive** | **CMV-IGRA weakly positive** | **CMV-IGRA positive** | **CMV-IGRA inconclusive** |
| --- | --- | --- | --- | --- | --- | --- |
| **Pre transplant (n=1,379)** |  |  | |  |  |  |
| R+ (n=1036) | 110 (11%) | | 35 (3%) | 15 (1%) | 875 (84%) | 1 (<1%) |
| D+/R- (n=232) | 232 (100%) | | 0 (0%) | 0 (0%) | 0 (0%) | 0 (0%) |
| D-/R- (n=111) | 108 (97%) | | 0 (0%) | 0 (0%) | 0 (0%) | 3 (3%) |
| **Eight weeks (n=1,195)** |  | |  |  |  |  |
| R+ (n=889) | 95 (11%) | | 28 (3%) | 12 (1%) | 749 (84%) | 5 (1%) |
| D+/R- (n=205) | 202 (99%) | | 1 (<1%) | 0 (0%) | 0 (0%) | 2 (1%) |
| D-/R- (n=101) | 101 (100%) | | 0 (0%) | 0 (0%) | 0 (0%) | 0 (0%) |
| **One year (n=997)** |  | |  |  |  |  |
| R+ (n=739) | 51 (7%) | | 23 (3%) | 5 (1%) | 659 (89%) | 1 (<1%) |
| D+/R- (n=170) | 97 (57%) | | 3 (2%) | 6 (4%) | 62 (36%) | 2 (1%) |
| D-/R- (n=88) | 83 (94%) | | 0 (0%) | 1 (1%) | 3 (3%) | 1 (1%) |

|  | **CMV-IGRA negative** | **CMV-IGRA grey-zone reactive** | **CMV-IGRA weakly positive** | **CMV-IGRA positive** |  |
| --- | --- | --- | --- | --- | --- |
| **Pre transplant CMV-IGRA negative**  **R+ (n=160)** | 110 (69%) | 35 (22%) | 15 (9%) | 0 (0%) | |
| **Eight weeks CMV-IGRA negative**  **R+ (n=135)** | 95 (70%) | 28 (21%) | 12 (9%) | 0 (0%) | |
| **One year**  **CMV-IGRA negative**  **R+ (n=79)** | 51 (65%) | 23 (29%) | 5 (6%) | 0 (0%) | |

**Table 2:** CMV-IGRA using categorical variables in R+ IGRA negative (</=0.4 IU INF-γ mL) kidney transplant recipients


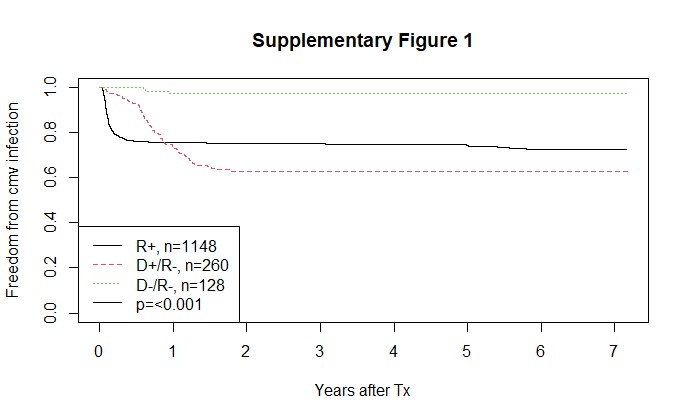


Kaplan-Meier analysis of freedom from CMV infection after kidney transplantation between CMV serostatus risk groups. All patients were subjected to weekly CMV DNAemia monitoring the first 2 months, monthly up to one year and on clinical indication afterwards. Only D+/R- patients received valganciclovir (900 mg once daily, adjusted to renal function) for 6 months. Monitoring of CMV were lacking in nine R+ recipients and one D-/R- recipient.


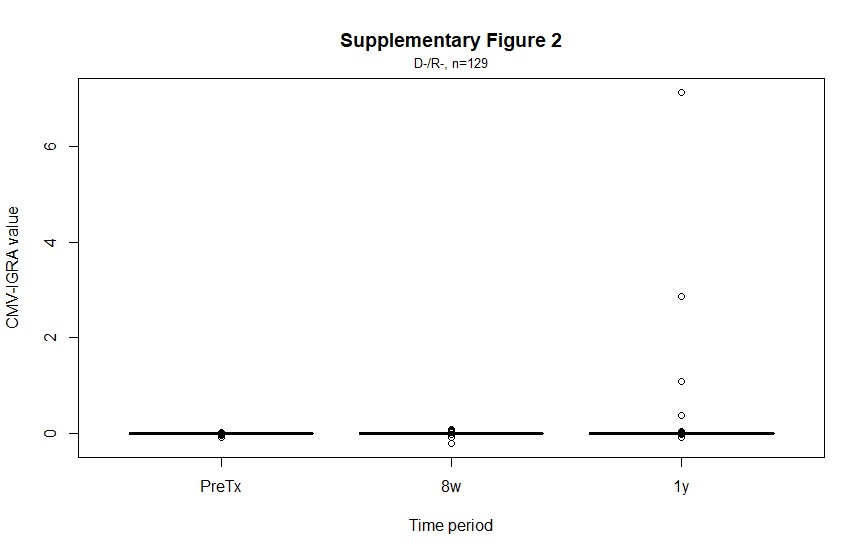


Box plot showing CMV-IGRA values among all CMV negative kidney recipients who received a CMV negative kidney (D-/R-) before transplantation, eight weeks post-transplantation and one year post-transplantation.
